# Severe hypotension induced by Almonertinib: a case report with literature review and clinical insights

**DOI:** 10.3389/fonc.2025.1566768

**Published:** 2025-07-03

**Authors:** Haiyu Niu, Feixue Song, Xiaochun Zhou, Qiaoying Jin, Yating Liu, Benxin Luo, Hanwen Wei

**Affiliations:** ^1^ Department of Oncology, The Second Hospital & Clinical Medical School, Lanzhou University, Lanzhou, China; ^2^ Department of Nephrology, The Second Hospital & Clinical Medical School, Lanzhou University, Lanzhou, China; ^3^ Cuiying Biomedical Research Center, The Second Hospital & Clinical Medical School, Lanzhou University, Lanzhou, China; ^4^ Department of Internal Medicine, The People’s Hospital of Zhouqu, Gannan, China; ^5^ Department of Cardiology, The First People’s Hospital of Lanzhou, Lanzhou, China

**Keywords:** lung adenocarcinoma, gastric adenocarcinoma, Almonertinib, hypotension, *EGFR*

## Abstract

Lung adenocarcinoma is a common malignancy in clinical practice, but the coexistence of lung and gastric adenocarcinomas in a single patient is rare. This report presents the case of a 70-year-old male with a history of smoking for over 30 years, diagnosed with both lung adenocarcinoma and gastric adenocarcinoma through lung biopsy and gastroscopy. Following comprehensive evaluations and exclusion of treatment contraindications, the patient underwent a therapeutic regimen comprising Sintilimab combined with nab-paclitaxel and cisplatin. Genetic testing of the lung cancer tissue identified mutations in the epidermal growth factor receptor (*EGFR*) gene, specifically p.L858R in exon 21 and p.T790M in exon 20. Consequently, the patient was prescribed Almonertinib at a dose of 110 mg/day to target these mutations. Approximately 72 h after initiating Almonertinib, the patient developed dizziness and nausea, accompanied by hypotension (blood pressure: 80/58 mmHg). Echocardiographic findings and NT-proBNP levels indicated no structural cardiac abnormalities or significant dysfunction. Almonertinib was discontinued, but subsequent attempts to reintroduce the drug consistently resulted in hypotension. After cardiology specialists evaluation, the hypotension was attributed to Almonertinib, prompting its permanent discontinuation. The treatment was adjusted to replace Almonertinib with Furmonertinib at a dose of 80 mg/day for lung adenocarcinoma, while maintaining the initial immunotherapy and chemotherapy regimen for gastric adenocarcinoma. Following these adjustments, the patient experienced no recurrence of hypotension. This case report reviews the literature to explore potential mechanisms of Almonertinib-induced hypotension and offers insights into the prevention, diagnosis, and management of similar adverse events in clinical practice.

## Introduction

Lung cancer remains one of the leading causes of cancer-related morbidity and mortality worldwide, with a rapidly increasing incidence. Approximately 75% of lung cancer patients are diagnosed at advanced stages, rendering them ineligible for surgical resection (1, 2). Based on histological characteristics, lung cancer is categorized into small cell lung cancer (SCLC) and non-small cell lung cancer (NSCLC), with NSCLC accounting for approximately 85% of cases (3). NSCLC is further classified into adenocarcinoma, squamous cell carcinoma, large cell carcinoma, and other subtypes, with lung adenocarcinoma being the most prevalent, constituting about 50% of cases (4, 5). *EGFR* mutations are among the most common driver mutations in NSCLC, occurring in about 28.2% of NSCLC cases in China and up to 50.2% in lung adenocarcinoma cases (6). Gastric adenocarcinoma, on the other hand, originates from the epithelial cells of the gastric mucosa, commonly affecting the inner lining of the stomach. As the disease progresses, cancer cells can infiltrate the stomach wall and metastasize to adjacent tissues or distant organs. Risk factors include *Helicobacter pylori* infection, genetic predisposition, and dietary habits (7). While surgical resection remains the primary treatment for early-stage gastric adenocarcinoma, advanced or metastatic cases often require chemotherapy combined with immunotherapy or targeted therapies, with radiation therapy employed in select situations (8, 9). Although various therapeutic strategies, such as surgery, chemotherapy, radiation therapy, targeted therapy, and immunotherapy, are utilized for lung adenocarcinoma and gastric adenocarcinoma, the coexistence of both malignancies in a single patient is rare. This rarity is compounded by the absence of standardized diagnostic and treatment protocols for such cases.

This report details the case of a 70-year-old patient diagnosed with concurrent lung and gastric adenocarcinoma, harboring *EGFR* mutations (L858R and T790M). During treatment with Almonertinib, the patient developed hypotension, which resolved after switching to Furmonertinib. Given the unusual nature of this case, it highlights the importance of recognizing and managing adverse drug reactions associated with targeted therapies. Furthermore, the case underscores the need for personalized treatment plans that consider a patient’s genetic profile and the potential side effects of therapeutic agents to optimize clinical outcomes. This report also contributes to the growing body of literature on managing toxicities related to *EGFR* mutation-targeted therapies, offering valuable insights for clinicians in the field.

## Case presentation

**Figure 1:**
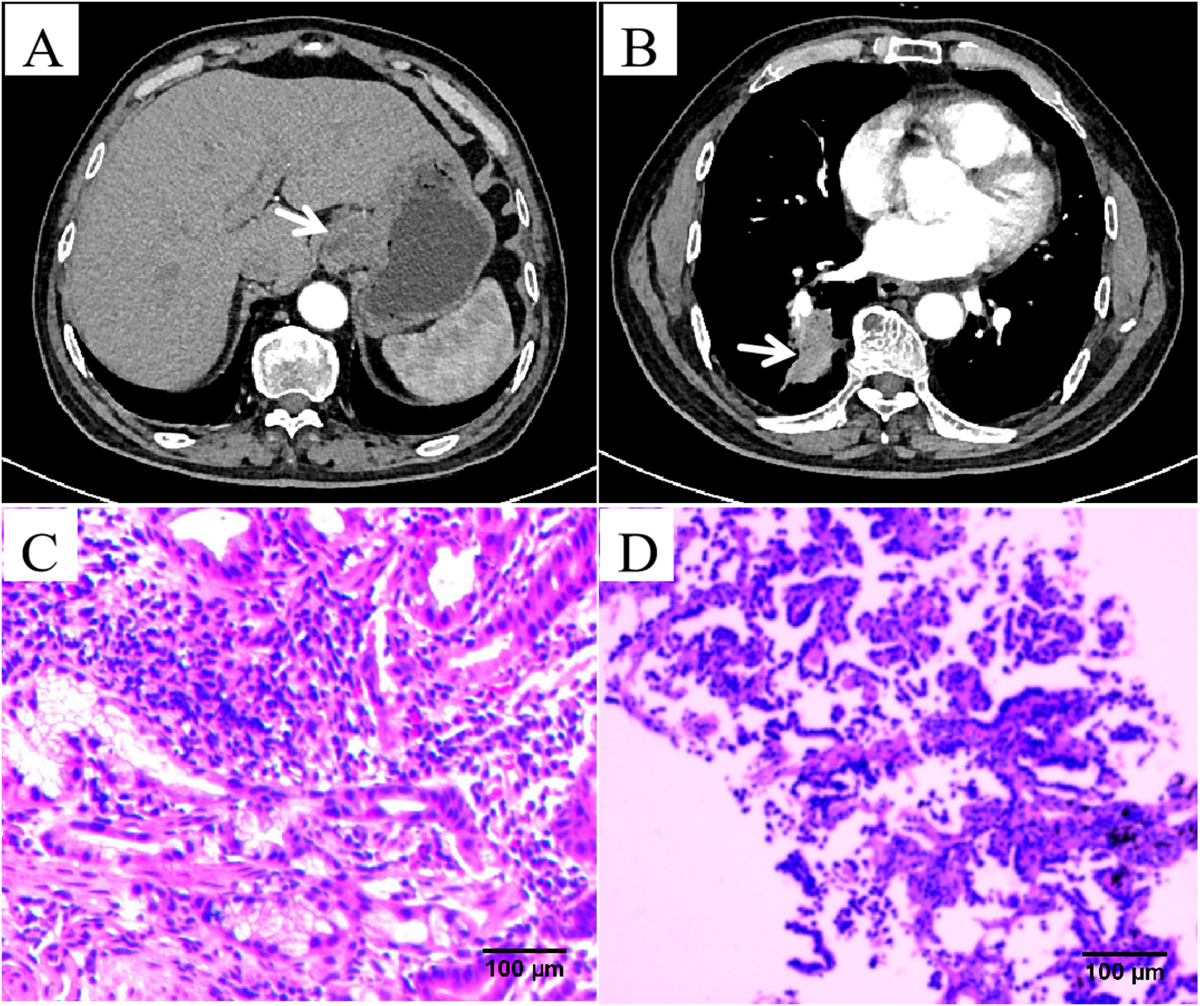
Imaging results from a 70-year-old male patient affected by both lung adenocarcinoma and gastric adenocarcinoma. **(A)** Abdominal computed tomography (CT) scan images revealed thickening of the gastric wall at the gastroesophageal junction and gastric fundus. **(B)** Chest CT scan images revealed a mass in the posterior segment of the right lower lung lobe. **(C)** Hematoxylin and eosin (HE) staining revealed glands with an irregular tubular structure, glandular epithelial cells showing large, hyperchromatic, atypical nuclei, and an increased nuclear-to-cytoplasmic ratio (×200). **(D)** HE staining revealed glandular structures with abnormal hyperplasia, pronounced nuclear atypia, and prominent nucleoli (×200).

A 70-year-old male with a history of smoking for over 30 years was admitted to our hospital in May 2023 with complaints of “dysphagia lasting over 1 month and a lung mass detected approximately 20 days ago.” The patient had no prior history of hypertension, type 2 diabetes mellitus, coronary artery disease, or other chronic conditions. There was no known history of allergies to food or medications. During the episodes of hypotension, the patient was administered Shengmai oral liquid (10 mL, three times daily), but the effect on blood pressure was limited. No other specific medications were used during these periods. Upon admission, chest and abdominal CT scans revealed the following findings: thickening of the gastric wall at the gastroesophageal junction and gastric fundus (**Figure 1A**), multiple slightly enlarged lymph nodes in the hepatogastric space, and suspected gastric cancer (preliminary staging: T4aN2M1). Endoscopic examination was recommended. Additional findings included multiple pulmonary nodules and a mass in the posterior segment of the right lower lung lobe (**Figure 1B**), suggestive of metastasis, a small cyst in segment IV of the liver, bilateral renal cysts, and prostatic calcification. Gastroscopy revealed a malignant tumor at the gastroesophageal junction and gastric fundus. Pathological examination of the biopsy confirmed gastric-type adenocarcinoma (**Figure 1C**). Immunohistochemistry results for the gastric tumor were as follows: CK8/18 (+), CKp (+), CD20 (-), CD56 (+), Syn (-), p53 (wild type), C-erbB-2 (0), CD34 (-), and Ki67 positivity in 70% of cells. A lung biopsy of the right lower lobe mass revealed adenocarcinoma (**Figure 1D**). Immunohistochemistry findings for the lung tumor included CKp (+), Napsin A (+), CK7 (+), TTF-1 (+), Syn (-), CD56 (-), CgA (-), P63 (-), CK5/6 (-), P40 (-), LCA (-), Villin (-), and Ki67 positivity in 10% of cells.

**Figure 2:**
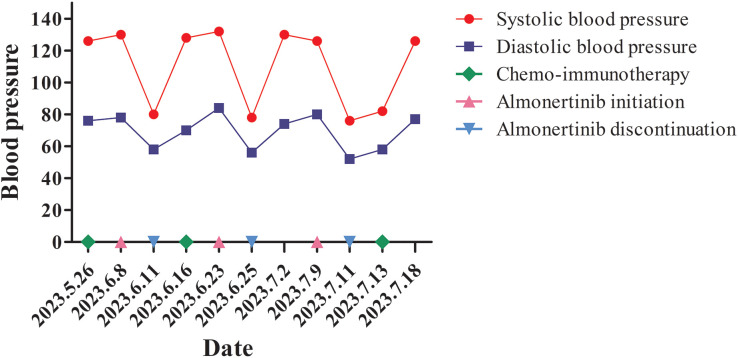
Blood pressure fluctuations in the present case.

Based on the concurrent diagnoses of lung and gastric adenocarcinoma, and after ruling out contraindications, the patient began treatment in May 2023. The regimen included Sintilimab (200 mg on day 1), nab-paclitaxel (400 mg on day 1), and cisplatin (40 mg on days 1–3), administered every 21 days. The treatment proceeded without complications. Genetic testing of the lung biopsy tissue, performed concurrently, revealed *EGFR* mutations in exon 21 (p.L858R) and exon 20 (p.T790M). Consequently, Almonertinib was administered as oral tablets, 110 mg once daily to target the identified EGFR mutations (10). However, approximately 72 h after starting Almonertinib, the patient developed dizziness and nausea, with a blood pressure reading of 80/58 mmHg, heart rate 90 bpm, temperature 36.6°C, respiratory rate 18 breaths/min, and SpO_2_ 94% on room air. Echocardiography showed a left ventricular ejection fraction of 63% with no structural abnormalities. Laboratory tests revealed a normal NT-proBNP level (86 pg/mL) and normal serum cardiac enzyme levels. Almonertinib was discontinued, and the patient’s blood pressure returned to 128/70 mmHg within 5 days. One week later, Almonertinib was reintroduced at the same dose. Approximately 48 h after resumption, the patient again experienced dizziness and nausea, with hypotension (blood pressure: 78/56 mmHg). Echocardiography demonstrated a left ventricular ejection fraction of 65%, with NT-proBNP levels at 78 pg/mL and normal serum cardiac enzymes. Almonertinib was discontinued, and blood pressure normalized to 130/74 mmHg after 1 week. A third attempt to resume Almonertinib (110 mg/day) was made 1 week later, but similar symptoms of dizziness and nausea reoccurred within 48 h, accompanied by hypotension. Almonertinib was permanently discontinued, and the patient’s blood pressure returned to normal within a week (blood pressure fluctuations are detailed in **Figure 2**). At the time of hypotension, the patient remained afebrile and clinically stable. Electrocardiograms were normal, with no arrhythmias or ischemic changes. As there were no signs of infection, sepsis workup and blood cultures were not performed. The patient did not develop tachycardia, rash, wheezing, diarrhea, or other symptoms suggestive of hypersensitivity or fluid loss.

During each episode of hypotension, laboratory evaluations including complete blood count, serum electrolytes, and renal function were performed. Results remained within normal or near-normal ranges across all three episodes (e.g., WBC: 4.9–5.8×10^9^/L; Hb: 135–143 g/L; PLT: 126–132×10^9^/L; sodium: 138–145 mmol/L; potassium: 3.8–4.5 mmol/L; creatinine: 75–80 μmol/L), without evidence of infection, electrolyte imbalance, or renal dysfunction. These findings suggest that the hypotension was unlikely related to chemotherapy or systemic metabolic disturbances. In addition, the patient was not receiving immune checkpoint inhibitors concurrently with EGFR-TKI therapy, thereby excluding potential synergistic vascular or cardiac effects. To date, no clinical trials have reported hypotension as a common adverse event associated with Almonertinib, indicating that this may represent a rare or under-recognized toxicity. A delayed immune-mediated reaction was also considered. Although typical Type I allergy was unlikely due to the timing and lack of rash or respiratory symptoms, the recurrent hypotension may reflect a non-immediate drug reaction. As noted by Rodríguez-Pérez et al., such reactions can be hard to diagnose without specialized testing (11). No allergy consultation was performed in this case.

Cardiology specialists evaluated the case and determined that the hypotension was associated with Almonertinib use. It was recommended to discontinue the drug. Subsequently, Almonertinib was replaced with Furmonertinib (80 mg/day) for the treatment of lung adenocarcinoma. The patient continued the initial treatment regimen of Sintilimab (200 mg on day 1), nab-paclitaxel (400 mg on day 1), and cisplatin (40 mg on days 1–3) every 21 days for gastric adenocarcinoma. Following these adjustments, no further episodes of hypotension occurred.

## Discussion

In 2022, global cancer statistics reported approximately 20 million new cases and 9.7 million cancer-related deaths (12). Lung cancer remained the leading cause of cancer incidence and mortality, accounting for 2.5 million new cases (12.4% of all newly diagnosed cancers) and 18.7% of all cancer-related deaths worldwide (12). With advances in genetic testing technologies, studies in NSCLC have demonstrated that molecularly targeted therapies based on driver gene mutations offer significant survival advantages compared to traditional chemotherapy, particularly therapies targeting mutations in *EGFR (* 13, 14). Among *EGFR* mutations, the exon 19 deletion (Del19) and the L858R point mutation in exon 21 are the most well-known sensitive mutations (15). First- and second-generation EGFR tyrosine kinase inhibitors (TKIs) have been shown to improve survival in NSCLC patients with these classic *EGFR* mutations (15). However, despite initial effectiveness, disease progression often occurs due to acquired resistance, most commonly caused by the secondary *EGFR* T790M mutation. This mutation significantly reduces the efficacy of first- and second-generation EGFR-TKIs (14, 16). Third-generation EGFR-TKIs, specifically designed to inhibit *EGFR* mutations, have become the treatment of choice for NSCLC patients with T790M mutations or disease progression following earlier-generation EGFR-TKIs. These third-generation inhibitors not only address T790M-mediated resistance but also outperform first-generation EGFR-TKIs in first-line treatment, prolonging progression-free survival (PFS) and overall survival (OS) (16).

Almonertinib, the first domestically developed third-generation EGFR-TKI in China, is specifically indicated for NSCLC patients with *EGFR* T790M mutations. This highly selective drug retains strong activity against both T790M mutations and classic *EGFR* mutations, such as exon 19 deletions and exon 21 L858R mutations (17, 18). Compared to earlier-generation EGFR-TKIs, Almonertinib has been shown to significantly extend median PFS while demonstrating a favorable safety and tolerability profile (19). A Phase III AENEAS trial conducted in China revealed that for patients with advanced NSCLC harboring EGFR-sensitive mutations, Almonertinib extended median PFS to 19.3 months, compared to 9.9 months with gefitinib (19). The duration of response was similarly extended to 18.1 months, compared to 8.3months with gefitinib. The incidence of adverse events remained relatively low (19).

In the case discussed, immunohistochemical staining and genetic profiling were crucial for differentiating between primary and metastatic lesions. Distinct immunohistochemical profiles confirmed the presence of two primary malignancies: lung adenocarcinoma and gastric adenocarcinoma. This rare coexistence presented significant challenges in diagnosis and treatment (20). Before obtaining the genetic testing results for the lung tumor, the patient was treated with Sintilimab, nab-paclitaxel, and cisplatin, a regimen effective for both lung and gastric adenocarcinomas. Genetic testing later identified the *EGFR* p.L858R mutation in exon 21 and the p.T790M mutation in exon 20. Given Almonertinib’s efficacy against both mutations, the patient was prescribed 110 mg daily. However, the patient developed hypotension shortly after starting Almonertinib, with blood pressure normalizing upon discontinuation. Normal echocardiographic findings and NT-proBNP levels ruled out cardiac dysfunction, suggesting that the hypotension was directly related to Almonertinib rather than a secondary cardiac event.

EGFR-TKIs have been associated with cardiotoxicity, including effects on cardiac ion channels. As noted by Li et al., some TKIs may block potassium channels such as hERG, potentially leading to QTc prolongation and arrhythmias (21). Although no QT prolongation or arrhythmia was observed in this patient, ion channel interference remains a possible off-target mechanism. Similar cases of hypotension associated with tyrosine kinase inhibitors (TKIs) have been reported. Tang et al. described a patient with EGFR-mutant NSCLC who developed hypotension shortly after afatinib administration, with no clear alternative cause identified (22). This case closely resembles ours in terms of clinical course and timing of symptom onset. Another report by Xiong et al. documented a savolitinib-induced reaction mimicking septic shock in a patient with advanced NSCLC, further highlighting the potential for TKIs to cause severe hemodynamic disturbances through mechanisms that remain poorly understood (23). These reports support the possibility that hypotension in our case may represent a rare, off-target adverse effect of Almonertinib.

Cardiologic evaluation indicated that the hypotension might result from non-cardiac mechanisms, potentially involving off-target effects on vascular smooth muscle, autonomic dysregulation, or drug-induced vasodilation. Although delayed hypotension following drug initiation is uncommon, differential diagnoses such as hypersensitivity reactions, adrenal suppression, or chemotherapy-related effects were considered unlikely due to the absence of related systemic symptoms and the clear temporal association with Almonertinib. While hypotension is a rare adverse effect of third-generation EGFR-TKIs, particularly Almonertinib, its occurrence in this case underscores the need for further investigation into its pharmacological mechanisms (24).

Furmonertinib, another third-generation EGFR-TKI with efficacy against T790M-positive NSCLC, was introduced as a replacement. The patient tolerated Furmonertinib without experiencing recurrent hypotension, highlighting differences in the side-effect profiles of EGFR-TKIs. This case emphasizes the importance of having alternative therapeutic options within the same drug class to manage severe or atypical adverse effects effectively.

Through multidisciplinary collaboration and individualized treatment adjustments, the patient successfully navigated adverse effects, and therapy proceeded without further complications. This case underscores the need for clinicians to remain vigilant for atypical adverse effects of EGFR-TKIs, such as hypotension, which may not be widely reported. The successful resolution of this patient’s condition by switching to Furmonertinib demonstrates the value of personalized treatment plans.

In conclusion, this report highlights the importance of monitoring cardiovascular complications in patients receiving EGFR-TKIs. Clinicians should remain alert to hypotension and other potential cardiovascular side effects, considering alternative EGFR-TKIs when necessary. Regular patient evaluations, multidisciplinary collaboration and individualized treatment adjustments are essential to ensure treatment safety and efficacy. Finally, further research is needed to elucidate the mechanisms underlying rare adverse reactions to EGFR-TKIs and identify predictive markers to guide clinical practice.

## Data Availability

The original contributions presented in the study are included in the article/supplementary material. Further inquiries can be directed to the corresponding author.
